# Surgery for lung adenocarcinoma with smokers’ polycythemia: a case report

**DOI:** 10.1186/1756-0500-6-38

**Published:** 2013-02-01

**Authors:** Yasoo Sugiura, Etsuo Nemoto, Hiromi Shinoda, Naoya Nakamura, Shizuka Kaseda

**Affiliations:** 1 National Hospital Organization, Kanagawa National Hospital, Pulmonary and Thoracic Surgery, 666-1 Ochiai, Hadano, Kanagawa, 257-8585, Japan; 2 Isehara Kyodo Hospital, Respiratory Medicine, 2-17-1 Sakuradai, Isehara, Kanagawa, 259-1132, Japan; 3 Tokai University Hospital, Division of Pathology, 143 Shimokasuya, Isehara, Kanagawa, 259-1193, Japan

**Keywords:** Smokers’ polycythemia, Phlebotomy, Predictive hematocrit, Lung cancer, Lung lobectomy

## Abstract

**Background:**

Smoking is a cause of cancer and polycythemia. Therefore, surgeons who treat patients with cancer may also encounter patients with polycythemia. However, few cases of surgical patients with polycythemia have been reported; in particular, a surgical case involving smokers’ polycythemia has never been reported. We herein report a patient with lung cancer and smokers’ polycythemia who successfully underwent lobectomy with control of hematocrit based on a modified formula in the perioperative period.

**Case presentation:**

A 67-year-old man underwent abdominoperineal resection for rectal carcinoma in June 2008. A ground glass opacity had been identified in the upper lobe of the right lung and was gradually enlarging. In March 2012, bronchoscopic cytology for investigation of the mass revealed non-small cell lung cancer, suggesting primary lung non-small cell carcinoma (T1bN0M0, Stage IA). When he was referred to our hospital for surgery, his complete blood count showed a red blood cell level of 6.50×10^6^/μL, hemoglobin of 21.0 g/dL, and hematocrit of 60.1%. The hematologists’ diagnosis was secondary polycythemia due to heavy smoking (smokers’ polycythemia) because the white blood cell and platelet counts were within normal limits and the erythropoietin was not increased. We calculated the appropriate phlebotomy and infusion volumes based on a formula that we modified. After 550 g of blood was phlebotomized to reduce the hematocrit to approximately 55%, video-assisted right lung upper lobectomy with lymph node dissection was performed in April 2012. The hematocrit was maintained at <50% postoperatively, and the patient was uneventfully discharged on postoperative day 7. The predictive hematocrit and measured hematocrit were very closely approximated in this case.

**Conclusion:**

We experienced a patient with smokers’ polycythemia who underwent right upper lobectomy for adenocarcinoma. The findings in this case report are meaningful for surgeons treating cancer patients because there are few reports discussing the perioperative care of surgical patients with polycythemia.

## Background

Smoking is a cause of cancer and polycythemia [1,2]. Therefore, surgeons who treat patients with cancer may encounter cases of polycythemia. However, few surgical patients with cancer and polycythemia have been reported. We experienced a patient who underwent lung lobectomy for lung cancer and had secondary polycythemia due to smoking. The most frequent complication of polycythemia is thrombosis [3]. We managed this patient’s hematocrit (HCT) to prevent thrombosis by performing phlebotomy and infusion based on a formula that we modified. As a result, the predictive HCT and measured HCT were very closely approximated, and we succeeded in providing effective perioperative care. This report provides useful information for the management of surgical patients with polycythemia.

## Case presentation

**Figure 1:**
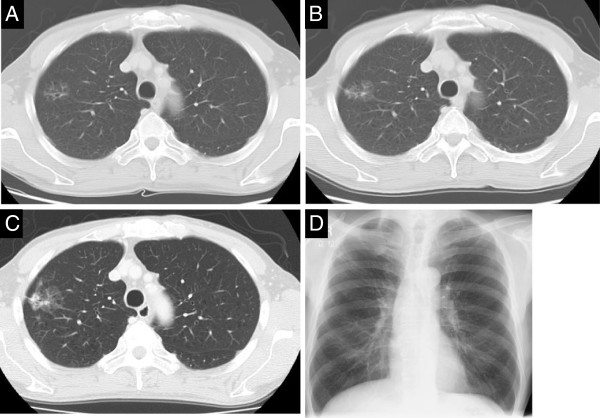
**Chest computed tomography and X-ray photography. **(**A**) Computed tomography, June 2009. (**B**) Computed tomography, January 2011. (**C**) Computed tomography, March 2012. (**D**) Chest X-ray photography, March 2012. The GGO at the lateral aspect of right S1 was enlarging, and it became a mass. GGO: ground glass opacity.

A 67-year-old man who underwent abdominoperineal resection for rectal carcinoma in June 2008 was evaluated for an abnormal chest shadow appearing as a ground glass opacity (GGO) in the right lung S1. It was gradually enlarging, and histology by brush cytology via bronchoscopy showed non-small cell lung cancer cells. He presented to our hospital for surgical treatment of the lung cancer (Figure 1).

His hypertension was being treated with 5 mg of amlodipine a day. He had been a three-packs-a-day smoker for 40 years, and he stopped smoking 4 weeks before surgery.

The blood examination on admission in March 2012 suggested polycythemia based on a red blood cell (RBC) count of 6.50×10^6^/μL, hemoglobin (Hb) of 21.0 g/dL, and HCT of 60.1%. Other blood cells and erythropoietin were not increased (Table 1). His complete blood count had been almost within normal limits until June 2009. Since September 2009, the RBC count, Hb, and HCT had been increasing (Table 2). The size of the GGO and its solid component had also been enlarging. The respiratory function examination showed obstructive impairment based on a vital capacity (VC) of 5.19 L, %VC of 151.3%, 1 second forced expiratory volume (FEV1.0) of 3.14 L, FEV1.0% of 63.7%, %FEV1.0 of 60.5% and diffusing capacity of the lung for carbon monoxide (DLCO) of 3.26 mL/min/mmHg/L.

**Table 1:** Blood examination results on admission in March 2012

					
TP	7.5 g/dL	WBC	8.2×10^3^/μL	PaCO_2_	39.8 mmHg
Alb	4.5 g/dL	RBC	6.50×10^6^/μL	PaO_2_	95.0 mmHg
AST/ALT	24/18 IU/L	Hb	21.0 g/dL	HCO_3_^-^	26.2 mmol/L
LDH	170 IU/L	HCT	60.1%	BE	1.9 mmol/L
T. Bil	1.4 mg/dL	MCV	92.5 fl	O_2_Hb	94.3%
ALP	367 IU/L	MCH	32.3 pg	COHb	2.3%
γ–GTP	102 IU/L	MCHC	34.9 g/dL	MetHb	0.5%
BUN/Cr	12.8/0.69 mg/dL	Plt	216×10^3^/μL	SaO_2_	97.0%
UA	9.0 mg/dL	Neut	65.3%		
Na/Cl/K	143/104/4.2 mEq/L	Lymph	15.6%		
T–Cho	184 mg/dL	Mono	14.2%		
		Eosino	4.0%		
CEA	7.4 ng/mL	Baso	0.9%		
ProGRP	60.6 pg/mL				
CYFRA	1.7 ng/mL				
Erythropoietin	8.8 mU/mL				
CCr	104.6 mL/min				

The blood gas was analyzed in room air. The reference value of erythropoietin is 8–36 mU/mL.

TP, total protein; Alb, albumin; AST, aspartate aminotransferase; ALT, alanine aminotransferase; LDH, lactase dehydrogenase; T. Bil, total bilirubin; ALP, alkaline phosphatase; γ–GTP, γ glutamyl transpeptidase; BUN, blood urea nitrogen; Cr, creatinine; UA, urinary acid; Na, sodium; Cl, chlorine; K, potassium; T–Cho, total cholesterol; CEA, carcinoembryonic antigen; ProGRP, pro–gastrin–releasing peptide; CYFRA, cytokeratin fragment 19; CCr, creatinine clearance; WBC, white blood cell; RBC, red blood cell; Hb, hemoglobin; HCT, hematocrit; MCV, mean corpuscular volume; MCH, mean corpuscular hemoglobin; MCHC, mean corpuscular hemoglobin concentration; Plt, platelet; Neut, neutrophil; Lymph, lymphocyte; Mono, monocyte; Eosino, eosinophil; Baso, basophil; PaCO_2_, arterial CO_2_ tension; PaO_2_, arterial O_2_ tension; HCO_3_^-^, bicarbonate ion; BE, base excess; O_2_Hb, oxyhemoglobin; COHb, carboxyhemoglobin; MetHb, methemoglobin; SaO_2_, arterial oxygen saturation.

**Table 2:** Course of CBC before surgery

	**June 2008**	**June 2009**	**Sep. 2009**	**Apr. 2010**	**Nov. 2010**	**June 2011**	**Feb. 2012**
Tumor size in maximum diameter (mm)		27				30	36
RBC (×10^6^/μL )	4.82	5.38	6.40	5.51	5.77	6.28	6.39
Hb (g/dL)	15.3	18.1	20.5	18.2	19.5	20.6	21.1
HCT (%)	44.0	50.2	58.2	52.7	54.3	57.2	60.8

RBC, red blood cell; Hb, hemoglobin; HCT, hematocrit.

Although a bone marrow examination was not performed, only one factor in the major and minor diagnostic criteria for polycythemia vera (PV) did not match [4,5]. In the major criteria, HCT was >60%, the cause of the secondary erythrocytosis was smoking, and splenomegaly was not present. In the minor criteria, the platelet count was <400×10^3^/μL, the neutrophil count was <12.5×10^6^/μL, and the serum erythropoietin level was within normal limits. Consultation with hematologists led us to conclude that these data were contradictive to a myeloproliferative disorder (e.g., chronic myeloid leukemia, polycythemia rubra vera, or essential thrombocythemia), and the diagnosis was smokers’ polycythemia (SP).

**Figure 2:**
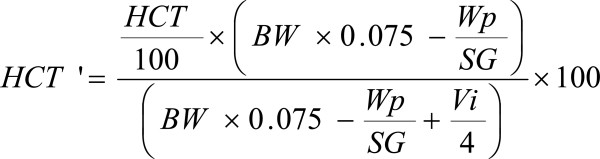
**Formula for predictive HCT after phlebotomy. **HCT: hematocrit, HCT': predictive HCT after phlebotomy, BW: body weight, Wp: weight of phlebotomy, SG: specific gravity, Vi: volume of infusion.

**Figure 3:**
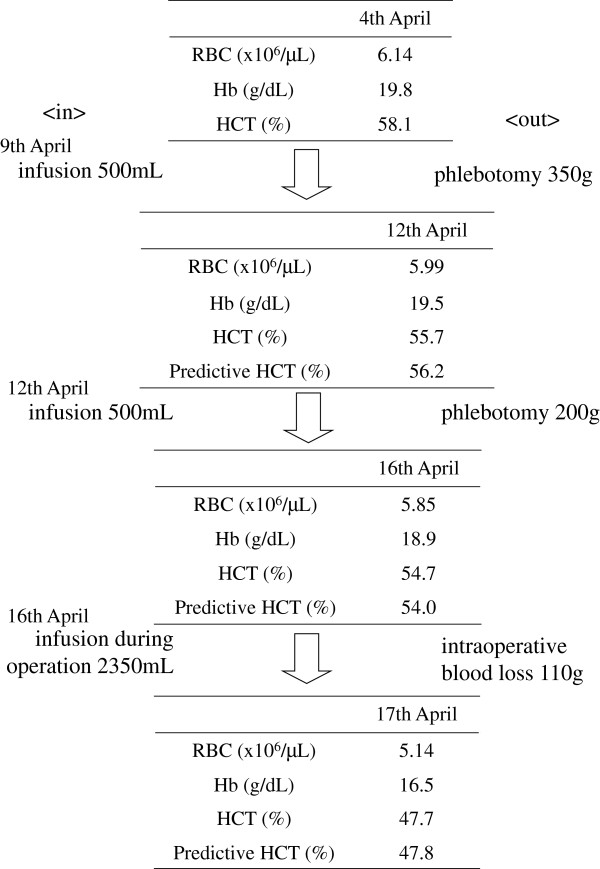
**Predictive HCT and phlebotomy in the perioperative period. **HCT: hematocrit.

We calculated the appropriate phlebotomy and infusion volumes based on a formula that we modified (Figure 2). A total of 350 and 200 g was phlebotomized 1 week and 4 days before the operation, respectively, to reduce the HCT to approximately 55%. Video-assisted right lung upper lobectomy and lymph node dissection were then performed in April 2012. The total intraoperative infusion volume was 2350 mL (7.8 mL/kg/hr), intraoperative urinary output was 500 mL, and intraoperative blood loss was 110 g (Figure 3). We maintained the HCT at <50%, and the patient was uneventfully discharged on postoperative day 7.

Pathological findings revealed that the bronchioalveolar carcinoma component occupied >90%. Immunohistologic staining for erythropoietin was negative. The pathological diagnosis was primary lung adenocarcinoma, 42×40×26 mm, pT2aN0M0 Stage IB (lymph node station 2, 0/1; 7, 0/4; 10, 0/7).

The serum erythropoietin level 1 month after discharge was normal at 11.2 mU/dL, as before the operation. However, the Hb and HCT in November 2012 were 22.0 g/dL and 61.0%, respectively.

## Conclusion

Smoking is one of the most notorious carcinogenic risk factors and a recognized cause of lung cancer [1]. Smoking is also a known cause of SP [2]. SP with hypertension is called Gaisböck syndrome and is frequently associated with cerebrovascular disease [6]. However, there are very few reports on surgery for patients with SP. Therefore, we reviewed the complications and treatments of PV [5,7-10]. According to the Polycythemia Vera Study Group, the most common cause of death in patients with polycythemia is thrombosis [3,10]. Thus, we formulated countermeasures to prevent the thrombosis during the perioperative period.

Cerebrovascular disease and high HCT in patients with SP are correlated, and treatments to prevent these complications include anticoagulants and/or antiplatelet agents and cytoreduction [3,11,12]. Thus, we chose to reduce the HCT before the operation because we had to be wary of using anticoagulants or antiplatelets during the perioperative period [13]. The optimal HCT is unclear in patients with SP, even in patients with PV [14], because, on theoretical grounds, erythrocytosis in this situation represents to a certain extent an “appropriate” physiological response to hypoxemia [15]. Thus, whatever the ideal HCT in cases of primary polycythemia, the corresponding ideal in cases of SP is likely to be higher [15]. We decided that the target HCT just before surgery was 55%, considering the average amount of intraoperative blood loss and for lobectomy in our hospital.

The total circulating blood volume is almost 1/13 (7.5%) of body weight, the RBC mass is 24 to 30 mL/kg, and the plasma volume is 39 to 49 mL/kg [16]. Approximately 25% of physiological saline remains in the vascular space, and the remaining 75% is distributed throughout the interstitial tissue [17]. We calculated the phlebotomy volume using the modified formula in Figure 2[18]. Determination of the predictive HCT and measured HCT in the perioperative period is shown in Figure 3.

The most important condition in this case was that homeostasis was maintained because of the presence of normal renal function and the fact that the patient underwent elective surgery. There are many methods and approaches to accurate calculation of the total circulating blood volume. However, this formula would be reliable if we had assumed that the body fluid, excluding the phlebotomy and infusion volumes, was in a stationary state. Practically, the predictive HCT and measured HCT were very closely approximated in this case.

It is unknown why the polycythemia became obvious after June 2008 and erythrocytosis developed 8 months after surgery. However, Weinberg et al. reported that significant increases in the prevalence and frequency of mutation of the *Janus kinase 2 (JAK2)* gene, the protein of which is especially important for controlling the production of blood cells from hematopoietic stem cells, may be related to poor DNA repair in smokers as a result of the increased demand for erythrocyte production [19]. *JAK2* mutation may have been present in this patient.

In conclusion, we experienced a patient with SP who underwent right upper lobectomy for adenocarcinoma. Smoking is a cause of cancer and polycythemia. Thus, this is a salutary case report for surgeons treating cancer patients because there are few reports discussing the perioperative care of surgical patients with polycythemia. This report is based on the experience of only one patient. Therefore, experience with surgical patients with SP should be accumulated and shared among surgeons to understand and improve treatment these cases.

## Consent

Written informed consent for the perioperative care and operation in this case was obtained on admission in accordance with institutional guidance based on the Helsinki Declaration. At the time of discharge, we reconfirmed that the patient gave permission to publish a case report about his clinical course. A copy of the written consent is available for review by the Editor-in-Chief of the journal.

## Abbreviations

GGO: Ground glass opacity; RBC: Red blood cells; Hb: Hemoglobin; HCT: Hematocrit; VC: Vital capacity; FEV 1.0: 1-second forced expiratory volume; DLCO: Diffusing capacity of the lung for carbon monoxide; SP: Smokers’ polycythemia; JAK2: Janus kinase 2.

## Competing interests

The author and co-authors have no potential conflict of interests to disclose. No financial or nonfinancial competing interests exist in this case report.

## Authors’ contributions

EN, SK, and YS operated on and treated this patient together in the perioperative period. HS determined the diagnosis by brush cytology via bronchoscopy as the primary physician. NN contributed to the pathological diagnosis and immunohistological chemistry of erythropoietin. SK was responsible for all aspects of management as president of Kanagawa National Hospital and chief of the Department of Pulmonary and Thoracic Surgery. All authors read and approved the final manuscript.
